# Supplementary material for: A Machine Learning Model to Predict Cardiovascular Events during Exercise Evaluation in Patients with Coronary Heart Disease
Source: J Clin Med. 2022 Oct 14;11(20):6061. doi: 10.3390/jcm11206061 (PMC9605581; doi:10.3390/jcm11206061)
Supplement: Supplementary file 1 [file jcm-11-06061-s001.zip › jcm-1904359-supplementary.pdf]

Supplementary Table S1. Importance ranking of clinical features included in machine learning.

	Feature name	Total score
1	Age	730
2	Duration of diabetes	662
3	Diabetes history	662
4	Myocardial infarction history	658
5	Male	642
6	VE/VCO_2slope	630
7	Smoking history	616
9	Hyperlipidemia history	552
8	VO_2@AT	564
10	Hypertension history	528
11	VO_2@AT /Pred	513
12	DBP@AT	511
13	VO_2peak	510
14	VEpeak	509
15	(VE/VCO_2peak)/Pred	497
16	VO_2peak/ Pred	490
17	$PETCO_2$ increase during exercise	489
18	HRpeak	482
19	Weight	478
20	O_2-pulse peak	473
21	Wpeak	461
22	HR increase during exercise	446
23	METs peak	445
24	ECG ST segment depression	438
25	SBP@AT	431
26	Height	431
27	RERpeak	424
28	RER@AT	424
29	Nitrates history	421
30	VE/VCO_2peak	421
31	SBPpeak	420
32	HRpeak/Pred	417
33	O_2-pulse peak/Pred	402
34	OUES	381

35	W@AT	363
36	HR@AT	362
37	Duration of hypertension	360
38	Exercise oscillatory ventilation	359
39	Resting HR	356
40	VE/VCO_2@AT	355
41	SBPpeak*HRpeak	353
42	Wpeak/Pred	349
43	DBPpeak	348
44	Resting $PETCO_2$	331
45	Resting VO_2	317
46	VE@AT	315
47	Statins history	311
48	VEpeak/Pred	309
49	METs@AT	294
50	VE/VCO_2@AT /Pred	284
51	Exercise habits	278
52	Employees	265
53	Antidiabetic drugs history	254
54	Duration of hyperlipidemia	253
55	Small airway dysfunction	241
56	Obstructive Ventilatory Dysfunction	237
57	Antiplatelet agent history	236
58	Family history of CVD	204
59	CCB history	202
60	Family history of CHD	202
61	Abnormal $PETCO_2$	201
62	BMI	199
63	Beta blocker history	192
64	ARB history	189
65	ACEI history	184
66	Abnormal O_2-pulse curve	182
67	Anticoagulants history	168
68	Known coronary heart disease	164
69	Exercise mode of ergometry cycle	159
70	Restrictive Ventilatory Dysfunction	152
71	Diuretics history	118

72	Digitalis history	108
73	Fibrates history	98

Abbreviation：VE/VCO_2slope, ventilation per carbon dioxide output slope; VO_2@AT, oxygen uptake at anaerobic threshold; VO_2@AT /Pred, ratio of oxygen uptake at anaerobic threshold to predicted; DBP@AT, diastolic blood pressure at anaerobic threshold; VO_2peak, peak oxygen uptake; VEpeak, peak ventilation; (VE/VCO_2peak)/Pred, ratio of peak ventilation per carbon dioxide output to predicted; VO_2peak/ Pred, ratio of peak oxygen uptake to predicted; $PETCO_2$, postapneic end-tial carbon dioxide pressure; HRpeak, peak heart rate; Wpeak, peak work rate; HR, heart rate; METs, metabolic equivalent; ECG, electrocardiogram; SBP@AT, systolic blood pressure at anaerobic threshold; RERpeak, peak respiratory exchange ratio; RER@AT, respiratory exchange ratio at anaerobic threshold; VE/VCO_2peak, peak ventilation per carbon dioxide output slope; SBPpeak, peak systolic blood pressure; HRpeak/Pred, peak heart rate to predicted; O_2-pulse peak/Pred, ratio of peak oxygen pulse to predicted; OUES, oxygen uptake efficiency slope; W@AT, work rate at anaerobic threshold; HR@AT, heart rate at anaerobic threshold; VE/VCO_2@AT, ventilation per carbon dioxide output at anaerobic threshold; Wpeak/Pred, ratio of peak work rate to predicted; DBPpeak, peak diastolic blood pressure; $PETCO_2$, postapneic end-tial carbon dioxide pressure; VE@AT, ventilation at anaerobic threshold; VEpeak/Pred, ratio of peak ventilation to predicted; METs@AT, work rate at anaerobic threshold; VE/VCO_2@AT /Pred, ratio of ventilation per carbon dioxide output at anaerobic threshold to predicted; CVD, cardiovascular disease; CCB, calcium channel blocker; CHD, coronary heart disease; BMI, body mass index; ARB, angiotensin receptor blockers; ACEI, angiotensin-converting enzyme inhibitor.

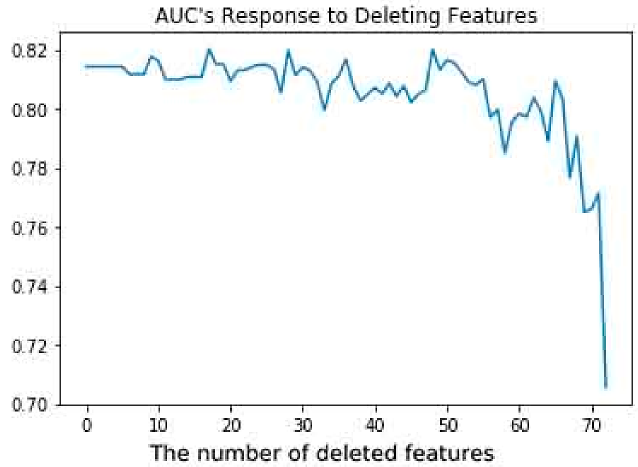

Supplementary Figure S1. AUC curve for feature selection. The curve of the test set shows that the obtained feature importance table can play a role in optimizing the results for a lightweight model.
